# A purification system for ^64^Cu produced by a biomedical cyclotron for antibody PET imaging

**DOI:** 10.1007/s10967-012-2340-7

**Published:** 2012-12-06

**Authors:** Teruaki Toyota, Tadashi Hanafusa, Takashi Oda, Iwane Koumura, Takanori Sasaki, Eiji Matsuura, Hiromi Kumon, Tsuneo Yano, Toshiro Ono

**Affiliations:** 1 Department of Radiation Research, Advanced Science Research Center, Okayama University, 2-5-1 Shikata-cho, Kita-ku, Okayama, 700-8558 Japan; 2 Graduate School of Health Sciences, Okayama University, 2-5-1 Shikata-cho, Kita-ku, Okayama, 700-8558 Japan; 3 Quantum Equipment Division, Sumitomo Heavy Industries, Ltd., 2-1-1 Osaki, Shinagawa-ku, Tokyo, 141-6025 Japan; 4 Collaborative Research Center for Okayama Medical Innovation Center, Graduate School of Medicine, Dentistry and Pharmaceutical Sciences, Okayama University, 2-5-1 Shikata-cho, Kita-ku, Okayama, 700-8558 Japan

**Keywords:** ^64^Cu, Cyclotron, Anion exchange chromatography, Positron emission tomography (PET), Atomic absorption spectrometry

## Abstract

Ion exchange is a simple and efficient method for separating no-carrier-added ^64^Cu from an irradiated Ni target. We developed a semi-automated two-round ^64^Cu separation system equipped with a strong-base anion exchange resin column. We first verified the efficiency of the system using a non-radioactive substitute consisting of 25 mg of Ni and 127 ng of Cu, and confirmed that Cu was completely eluted at the second round of the separation step. After the bombardment, separation of ^64^Cu from the Ni target was achieved with high radiochemical purity. ^64^Cu produced and separated in this study had an extremely low level of Ni impurity. It could be used for labeling monoclonal antibodies for antibody positron emission tomography imaging and synthesizing radiopharmaceuticals.

## Introduction

^64^Cu is a useful radionuclide for positron emission tomography (PET) [1, 2] as well as a potential radiation therapeutic reagent [3, 4], due to its intermediate half-life of 12.7 h and emission characteristics of both β^−^ (40 %) and β^+^ (19 %). ^64^Cu is normally produced from highly enriched ^64^Ni via the reaction of ^64^Ni (p, n) ^64^Cu by a cyclotron [5, 6]. For the separation of ^64^Cu from a ^64^Ni target and other trace amounts of byproducts, several methods can be used, such as precipitation, solvent extraction, electroplating, and ion exchange [7–9]. Among them, an ion exchange methodology using strong-base anion exchange resin is the most effective for the separation and purification of ^64^Cu [5, 10–12]. However, it is difficult to completely separate a tiny fraction of the cyclotron-produced ^64^Cu from the extremely large amount of the ^64^Ni target. For example, the ratio of ^64^Ni target to ^64^Cu is in the order of millions.

In the case of handling cyclotron-produced radioactive nuclides, we must avoid any manual performance, as this involves very high irradiation doses to the operators. Obata et al. [13] developed a remote-controlled ^64^Cu-separation apparatus equipped with a strong-base anion exchange resin column. In this study, we developed a semi-automated ^64^Cu-separation system, which is placed in the hot cell. It enabled the separation of high-quality and no-carrier-added ^64^Cu suitable for labeling monoclonal antibodies for antibody PET imaging.

## Materials and methods

### Reagent

Isotopically enriched ^64^Ni (99 %) was purchased from Isoflex Co. (San Francisco, CA, USA). Ultra-grade HCl and HNO_3_ were purchased from Sigma Aldrich (Tokyo, Japan). Cu and Ni standard solution (1 mg/ml) for atomic absorption spectrometry were obtained from Wako Pure Chemical Industries (Tokyo, Japan). Ultra-pure water was also from Wako Pure Chemical Industries.

### Preparation of Ni target and ^64^Cu production

The Ni target was prepared by the electrodeposition of enriched ^64^Ni on a 31-mm-diameter Au disk (Sumitomo Heavy Industries, Ltd., Tokyo, Japan). The Au disk, with the plated ^64^Ni (0.5 cm^2^), was mounted on a water-cooled target holder and irradiated with 12 MeV protons using a biomedical cyclotron (Cypris HM-12S, Sumitomo Heavy Industries, Ltd.). The production of ^64^Cu was performed at currents of 15–20 μA.

### Separation of ^64^Cu

After bombardment, ^64^Cu was separated from the Ni target in a single step on a strong-base anion exchange resin column using a prototype semi-automated separation apparatus (Sumitomo Heavy Industries, Ltd.). All of the solution was pumped and supplied to the column by N_2_ gas. The irradiated ^64^Ni was dissolved off the Au disk in 10 ml of 6 M HCl at 200 °C for 40 min and evaporated to dryness. The residue was dissolved in 10 ml of 6 M HCl and transferred onto a 0.8 × 4-cm AG1-X8 anion exchange column (Bio-Rad Laboratories, Inc., Hercules, CA, USA) equilibrated with 6 M HCl. The column was washed twice with 8 and 5 ml of 6 M HCl, and we collected ^64^Ni effluent for recycling. After switching the eluent to 10 ml of 0.1 M HCl, ^64^Cu was eluted and collected. ^64^Cu radioactivity of each eluate was measured in a dose calibrator (CRC-25PET, Capintec, Inc., Ramsey, NJ, USA).

### Non-radioisotope substitute for target-dissolved solution

The solution consisting of non-radioactive Ni and Cu was prepared to substitute for radioactive target-dissolved solution. A 101 mg of nickel chloride hexahydrate (25 mg of Ni) and 341 ng of copper chloride dihydrate (127 ng of Cu) were dissolved in 10 ml of ultra-grade nitric acid. The amount of Ni and Cu was described in a previous report [14].

### Atomic absorption spectrometry

A flame atomic absorption spectrometer (Z-9000, Hitachi, Ltd., Tokyo, Japan) equipped with a hollow cathode lamp was used for the determination of Ni and Cu. The wavelengths were 232.0 and 324.8 nm for Ni and Cu, respectively. Analytical working solution containing 100, 200, 400, and 800 ng of Ni and 12.5, 25, 50, and 100 ng of Cu were prepared by the appropriate dilution of the 1 mg/ml standard solution with ultra-grade nitric acid, respectively. The absorbance of blank, analytical solutions, and sample solutions was measured successively at the optimized operating conditions.

### Determination of radionuclide purity

The determination of radionuclide purity of separated ^64^Cu was performed by γ-spectrometry with a Ge semiconductor detector (GMX15P4-70, SEIKO EG&G, Tokyo, Japan). Data analysis was performed using Gamma Studio software (SEIKO EG&G).

## Results and discussion

Table 1 shows the results of two independent production of ^64^Cu. The separation of ^64^Cu from the Ni target was performed in a single-round anion exchange resin column using a prototype semi-automated apparatus. Nearly 50 % of ^64^Cu was eluted and separated. However, a significant amount of the ^64^Cu radioisotope went to Ni waste (column flow-through and effluent) and remained on the resin column. The results showed the poor reproducibility of the single-round separation methodology.

**Table 1:** ^64^Cu production and separation results by the prototype separation apparatus

	1	2
Bombardment		
Current (μA)	15	20
Time (min)	70	150
Charge (mC)	60.3	174.1
Yield (MBq/μA h)	33.5	28.6
Predicted yield (MBq/μA h)	36.8	35.3
Radioactivity (MBq)^a^		
Cu eluate	31.0 (47.3 %)	277 (52.8 %)
Ni effluent	9.4 (14.3 %)	244 (46.0 %)
Remaining in column	25.2 (38.4 %)	5.1 (1.1 %)

^a^Radioactivity was decay-corrected at the measurement after loading of the target-resolved solution onto the column according to the half-life of ^64^Cu (12.7 h)

**Fig. 1:**
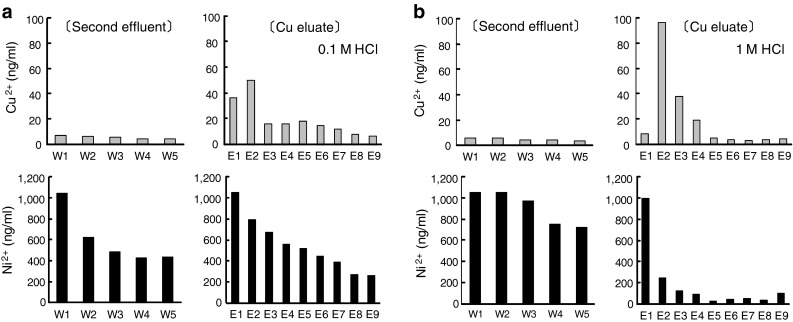
Insufficient separation performance by the prototype separation apparatus. Non-radioisotope substitute solution consisting of Ni and Cu was applied to the single-round prototype separation apparatus. Cu was eluted with 0.1 M HCl (**a**) or 1 M HCl (**b**) solution. A 1-ml fraction was collected at each step, and the Ni and Cu concentration was determined by the atomic absorption spectrometer

In order to investigate the separation efficiency of the single-round methodology, a non-radioisotope substitute consisting of 25 mg of Ni and 127 ng of Cu was applied to the prototype apparatus. A 1-ml fraction was collected at each step for Ni or Cu determination using the flame atomic absorption spectrometer. As shown in Fig. 1, Ni was not completely washed out even in the last washing step with 6 M HCl. A large amount of Ni remained on the column. Ni was continuously stripped from the column and mixed in the Cu eluate portion. Cu was efficiently eluted with 1 M HCl compared with 0.1 M HCl. However, twice the amount of Ni (ab. 200 ng) was contaminated even in the second 1-ml portion of the Cu eluate (ab. 100 ng).

**Fig. 2:**
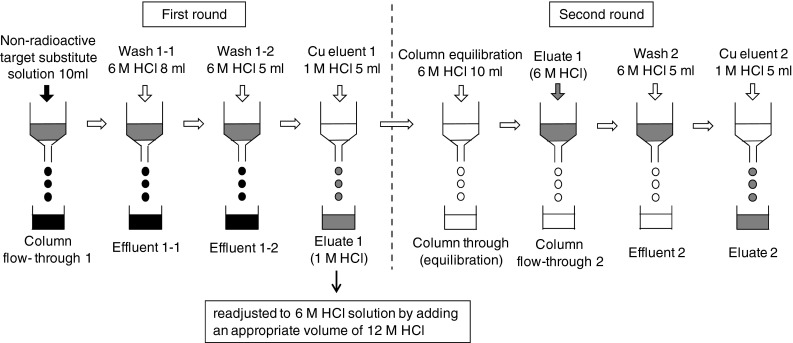
Schematic representation of the two-round separation methodology

**Fig. 3:**
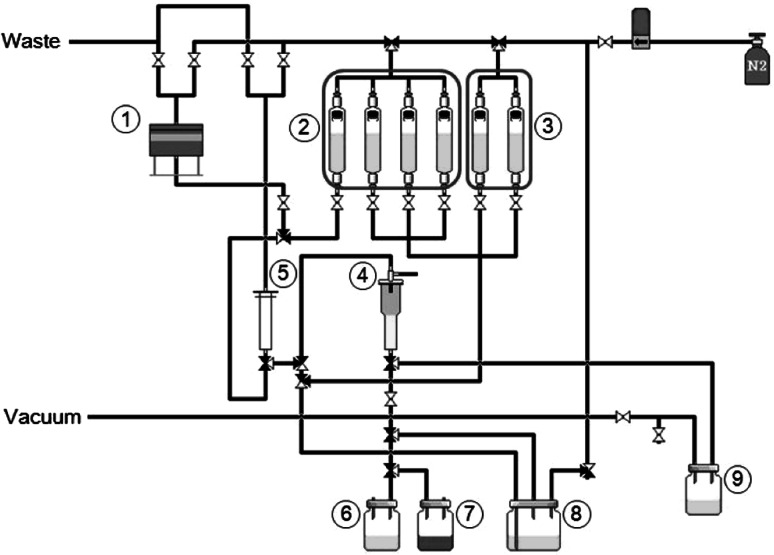
Two-round ^64^Cu separation system. (*1*) Vessel for target dissolving, (*2*) 6 M HCl washing solution vessel, (*3*) 1 M HCl washing solution vessel, (*4*) strong-base anion exchange resin column, (*5*) reservoir, (*6*) Ni collection vial (column flow-through), (*7*) vial for effluent, (*8*) vial for ^64^Cu eluate 1 (first round), (*9*) vial for ^64^Cu eluate 2 (second round)

**Fig. 4:**
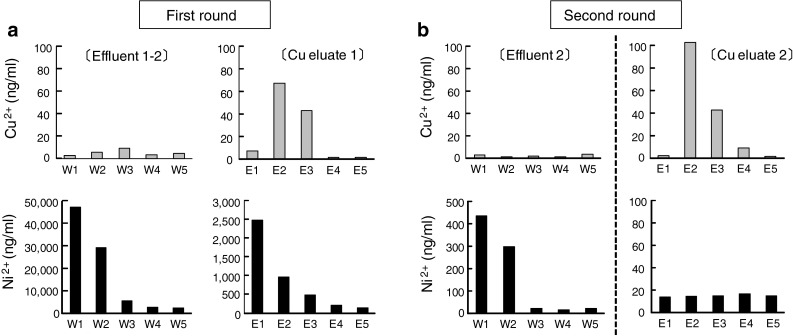
Separation and purification of Cu from Ni in a non-radioactive substitute. Non-radioactive substitute solution was applied to the two-round semi-automated separation system. A 1-ml fraction was collected at each step, and the Ni and Cu concentration was determined by the atomic absorption spectrometer. **a** First round of separation, **b** second (final) round of separation

Because of the insufficient separation performance of the single-round methodology, we developed the two-round separation methodology and constructed a new semi-automated separation apparatus (Figs. 2, 3). After the first round of column treatment, eluted and collected Cu in 1 M HCl solution was adjusted to 6 M HCl solution by adding an appropriate volume of 12 M HCl. It was further purified through the second round of column treatment (Fig. 2). All the solution was transferred to the column reservoir by N_2_ gas. Then, the solution was passed through the resin column from the reservoir at a normal atmospheric pressure (Fig. 3). We analyzed the Cu separation performance by this apparatus using a non-radioisotope substitute. A significant amount of Ni was still co-eluted with Cu at the first round of separation. However, by the second round of separation, a negligible amount of Ni was found in the Cu eluate (Fig. 4). Cu was completely eluted in the second to fourth fractions.

**Fig. 5:**
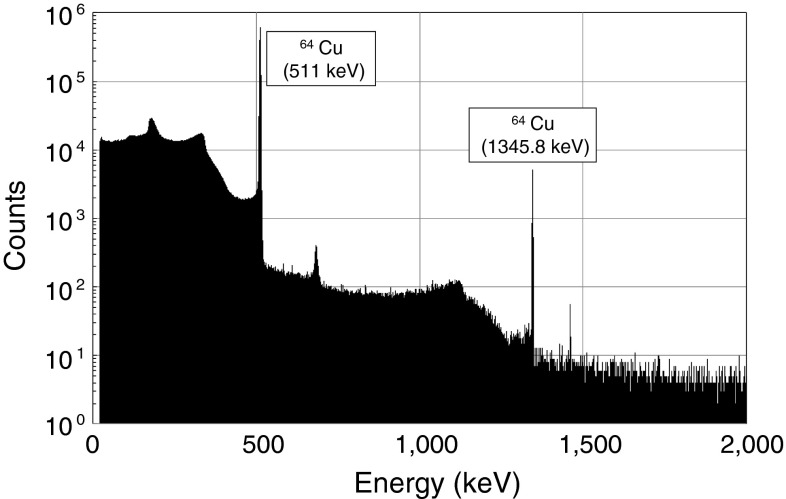
The γ-ray spectrum of the separated ^64^Cu fraction analyzed by a Ge semiconductor detector

We performed three independent ^64^Cu separations from a Ni target using the two-round semi-automated separation system. As shown in Table 2, 64–67 % of ^64^Cu was eluted by the first round of the separation step. In the subsequent second round of separation, 62–66 % of ^64^Cu was eluted and separated. This revealed that nearly 95 % of ^64^Cu eluted in the first round of separation was recovered in the second round of separation. A small amount of ^64^Cu was found in the column flow-through solution and effluent. A negligible amount of ^64^Cu remained on the resin column. With the use of the Ge detector, the radiochemical purity of the separated ^64^Cu was confirmed (Fig. 5).

**Table 2:** ^64^Cu separation results by the semi-automated two-round separation system

	1 (%)	2 (%)	3 (%)
First round			
Column flow-through 1	0.3^a^	0.4	0.1
Effluent 1-1	8.1	8.0	6.7
Effluent 1-2	16.2	14.8	13.9
Eluate 1	65.2	67.1	69.3
Second round			
Column through (equilibration)	0.8	0.7	0.6
Column flow-through 2	0	0.1	0
Effluent 2	1.3	2.4	1.6
Eluate 2	62.3	62.4	65.8
Remaining in column	0.9	0.8	0.4
Remaining in tube line	9.6	10.4	11.0

^a^They were decay-corrected at EOB according to the half-life of ^64^Cu (12.7 h), and expressed as a percentage

Ion exchange is a simple and efficient method for separating metals. It is based on the formation of anionic metal chloro complexes in a highly concentrated HCl solution and on the difference of their distribution coefficients in a strong-base anion exchange resin [15]. Ni cannot form a chloro complex in HCl solution and is not retained by the anion exchange resin. On the other hand, Cu can form a stable chloro complex in a concentrated HCl solution and is retained by the resin. After Ni is stripped from the resin column with a concentrated HCl solution, Cu can be successfully eluted from the resin column by an appropriate concentration of HCl. ^64^Cu is produced from highly enriched ^64^Ni via reactions of ^64^Ni (p, n) ^64^Cu by a cyclotron. Because the produced ^64^Cu is a very tiny fraction of the irradiation Ni target, it is usually difficult to obtain a high yield of ^64^Cu with extremely low Ni impurity. The remaining Ni will interfere with the antibody-labeling process of ^64^Cu. It may also be harmful to human health [16, 17].

Taken together, our semi-automated system enabled the separation of high-quality ^64^Cu suitable for labeling monoclonal antibodies for antibody PET imaging. In the current system, however, the target recovery at the end of bombardment and transfer to the hot cell has to be manually performed. A full-automated target handling system is currently being constructed to reduce exposure doses to the operators to as low as possible.

## Conclusion

In this study, we developed a semi-automated two-round ^64^Cu separation system. It is equipped with a strong-base anion exchange resin column. We first verified the efficient performance of the system, and confirmed that Cu was completely eluted at the second round of the separation step. There was a negligible amount of Ni in the Cu eluate. After the bombardment, separation of ^64^Cu from the Ni target was successfully achieved with high radiochemical purity. The semi-automated system enabled the separation of cyclotron-produced ^64^Cu suitable for labeling monoclonal antibodies for antibody PET imaging.
